# Classification of Circulating Tumor Cells by Epithelial-Mesenchymal Transition Markers

**DOI:** 10.1371/journal.pone.0123976

**Published:** 2015-04-24

**Authors:** Shiyang Wu, Suyan Liu, Zhiming Liu, Jiefeng Huang, Xiaoyu Pu, Jing Li, Dinghua Yang, Haijun Deng, Ning Yang, Jiasen Xu

**Affiliations:** 1 SurExam Bio-Tech, Guangzhou Technology Innovation Base, 80 Lan Yue Road, Science City, Guangzhou, P.R. China; 2 Oncology Department, Guangzhou General Hospital of Guangzhou Military Command, Guangzhou, P.R. China; 3 Department of Hepatobiliary Surgery, Guangzhou Nanfang Hospital, Guangzhou, P.R. China; 4 Department of General Surgery, Guangzhou Nanfang Hospital, Guangzhou, P.R. China; 5 Department of Pathology, The First Hospital Affiliated with Guangdong College of Pharmacy, Guangzhou, P.R. China; University of Alabama at Birmingham, UNITED STATES

## Abstract

In cancer, epithelial-mesenchymal transition (EMT) is associated with metastasis. Characterizing EMT phenotypes in circulating tumor cells (CTCs) has been challenging because epithelial marker-based methods have typically been used for the isolation and detection of CTCs from blood samples. The aim of this study was to use the optimized CanPatrol CTC enrichment technique to classify CTCs using EMT markers in different types of cancers. The first step of this technique was to isolate CTCs via a filter-based method; then, an RNA in situ hybridization (RNA-ISH) method based on the branched DNA signal amplification technology was used to classify the CTCs according to EMT markers. Our results indicated that the efficiency of tumor cell recovery with this technique was at least 80%. When compared with the non-optimized method, the new method was more sensitive and more CTCs were detected in the 5-ml blood samples. To further validate the new method, 164 blood samples from patients with liver, nasopharyngeal, breast, colon, gastric cancer, or non-small-cell lung cancer (NSCLC) were collected for CTC isolation and characterization. CTCs were detected in 107(65%) of 164 blood samples, and three CTC subpopulations were identified using EMT markers, including epithelial CTCs, biophenotypic epithelial/mesenchymal CTCs, and mesenchymal CTCs. Compared with the earlier stages of cancer, mesenchymal CTCs were more commonly found in patients in the metastatic stages of the disease in different types of cancers. Circulating tumor microemboli (CTM) with a mesenchymal phenotype were also detected in the metastatic stages of cancer. Classifying CTCs by EMT markers helps to identify the more aggressive CTC subpopulation and provides useful evidence for determining an appropriate clinical approach. This method is suitable for a broad range of carcinomas.

## Introduction

Most cancer-related deaths are associated with metastasis. Metastasis is a multi-step process with the presence of circulating tumor cells (CTCs) in the blood stream and disseminated tumor cells (DTCs) that home to the bone marrow [1]. CTCs disseminate from primary tumors by undergoing phenotypic changes that allow the cells to penetrate blood vessels [2, 3]. These changes are accompanied by a process described as epithelial-mesenchymal transition (EMT) [3], which is a complicated process that plays an essential role in metastasis [4]. EMT endows epithelial cells with enhanced invasive potential by the loss of their epithelial characteristics and the acquisition of a mesenchymal phenotype [5]. CTCs are a very heterogeneous population of cells, and one of the most common approaches for isolating CTCs is the epithelial cell adhesion molecule (EpCAM)-based enrichment technique. However, recent studies have demonstrated that this technique has failed to detect CTC subpopulations that have undergone EMT [6, 7]. These studies suggested that EMT markers could be used for the detection or capture of CTCs.

EMT is characterized by the downregulation of epithelial markers, such as EpCAM and cytokeratins (CK), and the upregulation of mesenchymal markers, such as vimentin and twist [8, 9]. EpCAM is a transmembrane glycoprotein that mediates cell-cell adhesion in epithelial tissues, and this protein has oncogenic potential via its capacity to upregulate c-myc, cyclin A and cyclin E [10]. CKs are the proteins of keratin-containing intermediate filaments found in the cytoskeleton of epithelial cells. Both EpCAM and CK are commonly used biomarkers for CTCs from epithelial-derived neoplasms [11, 12]. Vimentin, a member of the intermediate filament family of proteins, is ubiquitously expressed in mesenchymal cells [13], and expressing vimentin in cancer cells increases tumor growth and invasiveness [14]. Vimentin expression is associated with the upregulation of N-cadherin [15], and a previous study has demonstrated that the overexpression of vimentin in breast cancer is related to a poor prognosis [16]. Twist is a helix-loop-helix protein that is transcriptionally active during cell differentiation [17], and increased expression of twist has been observed in many types of tumor cells, such as prostate, gastric and breast cancer [18]. Furthermore, twist can repress E-cadherin and upregulate N-cadherin [19], and expressing twist in breast cancer cells results in resistance to paclitaxel [20].

Recently, studies have shown that EMT markers are expressed in CTCs in breast and hepatocellular carcinomas [21, 22]. The study by Yu et al. has provided evidence that CTCs exhibit dynamic changes in epithelial and mesenchymal composition. Mesenchymal CTCs are associated with metastasis and resistance to chemotherapy [7]. All of these data support EMT as a potential biomarker for the characterization of CTCs. In a previous study, we developed a CanPatrol CTC enrichment technique that combined a CD45 magnetic bead separation method and a filter-based method for CTC isolation [23]. However, the heterogeneity of CTCs and characteristics of blood samples from some cancer patients limited its broad clinical application. Therefore, in the present study, we attempted to optimize the CanPatrol CTC enrichment technique by removing the CD45 magnetic bead separation steps and using a more sensitive method to label the CTCs. We also investigated the feasibility of using epithelial and mesenchymal markers (EpCAM, CK8/18/19, vimentin and twist) to characterize and classify CTCs into three subpopulations, including epithelial CTCs, biophenotypic epithelial/mesenchymal CTCs, and mesenchymal CTCs. The expression of these molecules was investigated in the CTCs from patients with liver, nasopharyngeal, gastric, breast, or colon cancer or non-small-cell lung cancer (NSCLC).

## Materials and Methods

### Patient samples

Patients were recruited by the Guangzhou General Hospital of Guangzhou Military Command and Guangzhou Nanfang Hospital from July 2013 to June 2014. The purpose of this recruitment and sample collection was to classify CTCs by EMT markers using the optimized CanPatrol CTC enrichment technique (SurExam, Guangzhou, China) in different types of cancers. A total of 164 patients who were diagnosed with NSCLC or liver, nasopharyngeal, breast, colon or gastric carcinoma (29 with NSCLC, 40 with liver cancer, 24 with nasopharyngeal cancer, 18 with breast cancer, 38 with colon cancer, and 15 with gastric cancer) were recruited into this study (Table 1). Twenty-seven healthy volunteers were included as controls. For the cancer patients, peripheral blood samples (5 ml, anticoagulated with EDTA) were collected after discarding the first 2 ml to avoid potential skin cell contamination from the venipuncture. All blood samples were collected before surgery or other treatment. Among the patients, 10 NSCLC and 8 breast cancer patients volunteered to donate an additional 5 ml of blood to compare the efficacy of the CanPatrol CTC enrichment technique before and after optimization. From the healthy volunteers, 10ml blood samples were collected and used as negative controls or for spiking experiments. The blood samples were processed within 4 h of collection. This study was approved by the ethical committee of Guangzhou General Hospital of Guangzhou Military Command and Guangzhou Nanfang Hospital. Written informed consent was obtained from all the cancer patients and healthy volunteers in this study.

**Table 1:** Information and clinical characteristics of the patients.

	Liver cancer	Nasopharyngeal cancer	NSCLC	Breast cancer	Colon cancer	Gastric cancer
No. of patients	40 (100%)	24 (100%)	29 (100%)	18 (100%)	38(100%)	15(100%)
Age						
Range	28–77	29–61	34–76	31–63	22–69	32–71
Median	48	45	58	47	57	60
Sex						
Males	30 (75%)	14 (58%)	23 (79%)	0 (0%)	26 (71%)	15(100%)
Females	10 (25%)	10 (42%)	6 (21%)	18 (100%)	12(29%)	0 (0%)
Differentiation						
Well and moderate	31(78%)	10(42%)	11(38%)	12(67%)	26(68%)	3(20%)
Poor	9(22%)	14(58%)	18(62%)	6(33%)	12(32%)	12(80%)
Stage						
T1N0M0	11(28%)	0(0%)	0(0%)	0(0%)	0(0%)	0(0%)
T3N0M0	14(35%)	5 (21%)	3 (10%)	0(0%)	8 (21%)	0(0%)
T2N1M0	0(0%)	10 (42%)	0(0%)	12 (67%)	20 (53%)	0(0%)
T3N1M0	5(12%)	0(0%)	0(0%)	0(0%)	0(0%)	0(0%)
T3N2M0	0(0%)	0(0%)	6 (21%)	0(0%)	0(0%)	0(0%)
T3N1M1	10(25%)	9 (37%)	0(0%)	0(0%)	10 (26%)	7(47%)
T2N2M1	0(0%)	0(0%)	20(69%)	0(0%)	0(0%)	0(0%)
T3N2M1	0(0%)	0(0%)	0(0%)	6 (33%)	0(0%)	8(53%)

### Cell lines and cell culture

The HepG2 cell line (ATCC, HB 8065, derived from a human hepatocellular carcinoma) was used in this study. Cells were cultured in RPMI 1640 Medium (Thermo Fisher, Waltham, USA) supplemented with 10% fetal bovine serum (FBS) (Thermo Fisher, Waltham, USA) and 1% penicillin-streptomycin (Thermo Fisher, Waltham, USA) at 37°C in a CO_2_ incubator (Thermo Fisher, Waltham, USA) with 5% CO_2_.

### Isolation of CTCs by size

A filtration method was applied using a calibrated membrane with 8-μm diameter pores (Millipore, Billerica, USA). The required filtration system consisted of a filtration tube containing the membrane (SurExam, Guangzhou, China), a manifold vacuum plate with valve settings (SurExam, Guangzhou, China), an E-Z 96 vacuum manifold (Omega, Norcross, USA), and a vacuum pump (Auto Science, Tianjin, China). Erythrocytes were removed using a red blood cell lysis buffer (154 mM NH_4_Cl, 10 mM KHCO_3_ and 0.1 mM EDTA (all from Sigma, St. Louis, USA) in deionized water), then the remaining cells were resuspended in PBS (Sigma, St. Louis, USA) containing 4% formaldehyde (Sigma, St. Louis, USA) for 5 minutes before filtration. After the cell suspension was transferred to the filtration tube, the pump valve was switched on to reach at least 0.08MPa; the manifold vacuum plate valve was then switched on, and filtration began.

### Tri-color RNA in situ hybridization (ISH) assay

The RNA-ISH method that was applied in this study was based on the branched DNA (bDNA) signal amplification technology [26]. The bDNA signal amplification technology does not rely on *in vitro* amplification of a target sequence as PCR does. Instead, the sensitivity of this technology is achieved by signal amplification on a bDNA probe after direct binding of capture probes to the target sequences [26]. This technique uses a multi-step nucleic acid hybridization platform in which the target sequences are captured by multiple specific probes (known as capture probes), followed by conjugation to the bDNA signal amplification probes, which consist of three types of probes, including the preamplifier sequence, the amplifier sequence and the label probe. The preamplifier sequence is designed to hybridize to contiguous regions on the capture probes, and the other regions on the preamplifier are designed to hybridize to multiple bDNA amplifier sequences, creating a branched structure. Finally, the label probes conjugated to a fluorescent dye are complementary to the bDNA amplifier sequences. The label probes then bind to the bDNA molecule by hybridization. The capture probes sequences for the EpCAM, CK8/18/19, vimentin, twist, and CD45 genes and the sequences for the bDNA signal amplification probes are listed in Tables 2 and 3. All sequences were synthesized by Invitrogen (Invitrogen, Shanghai, China).

**Table 2:** Capture probe sequences for the EpCAM, CK8/18/19, vimentin, twist, and CD45 genes.

Gene	Sequences(5’→3’)
EpCAM	TGGTGCTCGTTGATGAGTCA
	AGCCAGCTTTGAGCAAATGA
	AAAGCCCATCATTGTTCTGG
	CTCTCATCGCAGTCAGGATC
	TCCTTGTCTGTTCTTCTGAC
	CTCAGAGCAGGTTATTTCAG
CK8	CGTACCTTGTCTATGAAGGA
	ACTTGGTCTCCAGCATCTTG
	CCTAAGGTTGTTGATGTAGC
	CTGAGGAAGTTGATCTCGTC
	CAGATGTGTCCGAGATCTGG
	TGACCTCAGCAATGATGCTG
CK18	AGAAAGGACAGGACTCAGGC
	GAGTGGTGAAGCTCATGCTG
	TCAGGTCCTCGATGATCTTG
	CAATCTGCAGAACGATGCGG
	AAGTCATCAGCAGCAAGACG
	CTGCAGTCGTGTGATATTGG
CK19	CTGTAGGAAGTCATGGCGAG
	AAGTCATCTGCAGCCAGACG
	CTGTTCCGTCTCAAACTTGG
	TTCTTCTTCAGGTAGGCCAG
	CTCAGCGTACTGATTTCCTC
	GTGAACCAGGCTTCAGCATC
Vimentin	GAGCGAGAGTGGCAGAGGAC
	CTTTGTCGTTGGTTAGCTGG
	CATATTGCTGACGTACGTCA
	GAGCGCCCCTAAGTTTTTAA
	AAGATTGCAGGGTGTTTTCG
	GGCCAATAGTGTCTTGGTAG
Twist	ACAATGACATCTAGGTCTCC
	CTGGTAGAGGAAGTCGATGT
	CAACTGTTCAGACTTCTATC
	CCTCTTGAGAATGCATGCAT
	TTTCAGTGGCTGATTGGCAC
	TTACCATGGGTCCTCAATAA
CD45	TCGCAATTCTTATGCGACTC
	TGTCATGGAGACAGTCATGT
	GTATTTCCAGCTTCAACTTC
	CCATCAATATAGCTGGCATT
	TTGTGCAGCAATGTATTTCC
	TACTTGAACCATCAGGCATC

**Table 3:** Sequences for the bDNA signal amplification probes.

	Function (copies)	Sequence(5'→3')	Complement
bDNA probes for EpCAM and CK8/18/19	capture probe tail(1)	CTACAAACAAACAATATT	preamplifier leader(1)
	preamplifier repeat(5)	CGCAGCCTCAGCC	amplifier leader(1)
	amplifier repeat(5)	CCCAGACCCTACC	label probe(1)
bDNA probes for vimentin and twist	capture probe tail(1)	CTTCTCAATAACTAACAT	preamplifier leader(1)
	preamplifier repeat(5)	GACGGTCGGCGTT	amplifier leader(1)
	amplifier repeat(5)	GTCACCGCTCCAC	label probe(1)
bDNA probes for CD45	capture probe tail(1)	CTTTATACCTTTCTTTCA	preamplifier leader(1)
	preamplifier repeat(5)	GCGCGCTGTAGGG	amplifier leader(1)
	amplifier repeat(5)	AGGCGAGGGGAGA	label probe(1)

The sequences labeled “leader” appear once in the indicated construct, while sequences labeled “repeat” appear the indicated number of times. The tail on the capture probe is a single sequence.

The assay was performed in a 24-well plate (Corning, NY, USA), and the cells on the membrane were treated with a protease (Qiagen, Hilden, Germany) before hybridization with capture probes specific for the epithelial biomarkers EpCAM and CK8/18/19, the mesenchymal biomarkers vimentin and twist, and the leukocyte biomarker CD45(Sequences are shown in Table 2). The hybridization was performed at 42°C for 2 hours, and the un-bound probes were then removed by washing three times with 1,000μl of wash buffer (0.1×SSC (Sigma, St. Louis, USA)). The signal amplification step was performed by incubating the sample with 100μl of preamplifier solution (30% horse serum(Sigma, St. Louis, USA), 1.5% sodium dodecyl sulfate(Sigma, St. Louis, USA), 3 mM Tris-HCl (pH 8.0) (Sigma, St. Louis, USA), and 0.5 fmol of preamplifier (the sequences are shown in Table 3) at 42°C for 20 minutes. The membranes were cooled, washed three times with 1,000μl of wash buffer (0.1×SSC), and then incubated with 100μl of amplifier solution(30% horse serum, 1.5% sodium dodecyl sulfate, 3 mM Tris-HCl(pH 8.0), and 1 fmol of amplifier (the sequences are shown in Table 3). Three types of fluorescently labeled probes (the sequences are shown in Table 3), which had been conjugated with the fluorescent dyes Alexa Fluor 594 (for the epithelial biomarkers EpCAM and CK8/18/19), Alexa Fluor 488(for the mesenchymal biomarkers vimentin and twist), and Alexa Fluor 647(for the leukocyte biomarker CD45), were added and incubated at 42°C for 20 minutes. After washing with 0.1×SSC, the cells were stained with 4′,6-diamidino-2-phenylindole (DAPI) (Sigma, St. Louis, USA) for 5 minutes and analyzed with a fluorescence microscope using a 100x oil objective (Olympus BX53, Tokyo, Japan).

### Spiking experiments

To study the recovery of the CTCs, the HepG2 cell line was used. The cells were harvested and washed with PBS containing 2 mM EDTA (Sigma, St. Louis, USA). The cells were counted and diluted to 1 cell/2 μl; 10, 50, 100 and 200 HepG2 cells were then spiked into 5 ml of blood from the healthy volunteers to analyze the recovery of the tumor cells. The assays were repeated 8 times for each number of the spiked cells. After red blood cell lysis, filtration, and RNA-ISH, the cells were counted with a fluorescence microscope using a 100x oil objective (Olympus BX53, Tokyo, Japan).

### Comparison of the efficacy of the CanPatrol CTC enrichment technique before and after optimization

Eighteen samples (10 samples from NSCLC patients and 8 samples from breast cancer patients) were used to compare the efficacy of the CanPatrol CTC enrichment technique before and after optimization. For each sample, 5 ml of blood was used for CTC isolation and characterization using each method. Before optimization, a combination of the CD45+ magnetic bead separation and filtration methods was used for CTC isolation, and an immunostaining method was applied for CTC characterization. The protocol of this method has been described before [23]. To classify CTCs using EMT biomarkers, an antibody cocktail consisting of anti-EpCAM (R&D, Minneapolis, USA), anti-CK8/18/19 (R&D, Minneapolis, USA), anti-vimentin (BD Bioscience, San Jose, USA), anti-twist (BD Bioscience, San Jose, USA) and anti-CD45 (Surexam, Guangzhou, China) was used to stain the CTCs.

## Results

### EpCAM, CK8/18/19, vimentin and twist expression in HepG2 cells and the blood leukocytes of healthy donors

**Fig 1:**
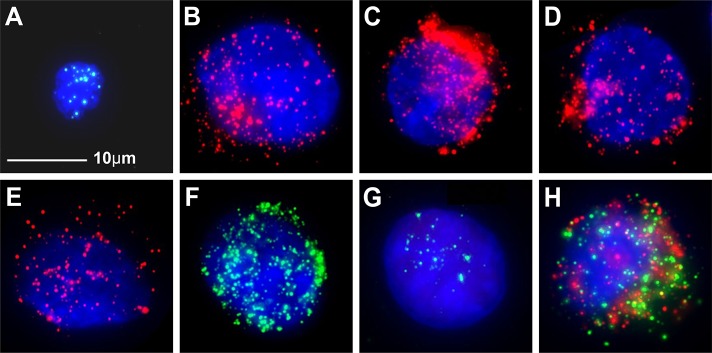
EpCAM, CK8/18/19, vimentin and twist expression in HepG2 tumor cells and leukocytes. **A**: negative control, leukocytes stained for CD45 expression (bright blue fluorescence); **B**: HepG2 cells stained for EpCAM expression (red fluorescence); **C**: HepG2 cells stained for CK8 expression(red fluorescence); **D**: HepG2 cells stained for CK18 expression(red fluorescence); **E**: HepG2 cells stained for CK19 expression(red fluorescence); **F**: HepG2 cells stained for vimentin expression (green fluorescence); **G:** HepG2 cells stained for twist expression(green fluorescence); **H:** HepG2 cells stained for EpCAM, CK8/18/19, vimentin and twist expression (red/green fluorescence). The cells were analyzed using a 100x oil objective

HepG2 cells spiked into 5 ml of blood from the healthy volunteers and processed as per the patient samples were used as positive controls for the detection of EpCAM, CK8/18/19, vimentin and twist (Fig 1). EpCAM, CK8/18/19, vimentin and twist expression was also investigated in the leukocytes from 20 healthy blood donors. Among these six biomarkers, only vimentin was expressed in some leukocytes. CD45 was expressed in leukocytes but not in tumor cells. No epithelial marker-positive or biophenotypic epithelial/mesenchymal marker-positive cells were found in the leukocytes. Therefore, the leukocytes were characterized as CD45^+^DAPI^+^ or vimentin^+^CD45^+^DAPI^+^ cells. The tumor cells were epithelial marker-positive CD45^-^DAPI^+^ cells, biophenotypic epithelial/mesenchymal marker-positive CD45^-^DAPI^+^, or mesenchymal marker-positive CD45^-^DAPI^+^ cells.

### Efficiency of tumor cell recovery

To study the efficiency of tumor cell recovery using this technique, 10, 50, 100 and 200 HepG2 cells were spiked into 5 ml of blood to analyze the recovery of the tumor cells. The assays were repeated 8 times at each number of spiked HepG2 cells.

The results demonstrated that the enrichment process was linear (R^2^ = 0.999).

**Fig 2:**
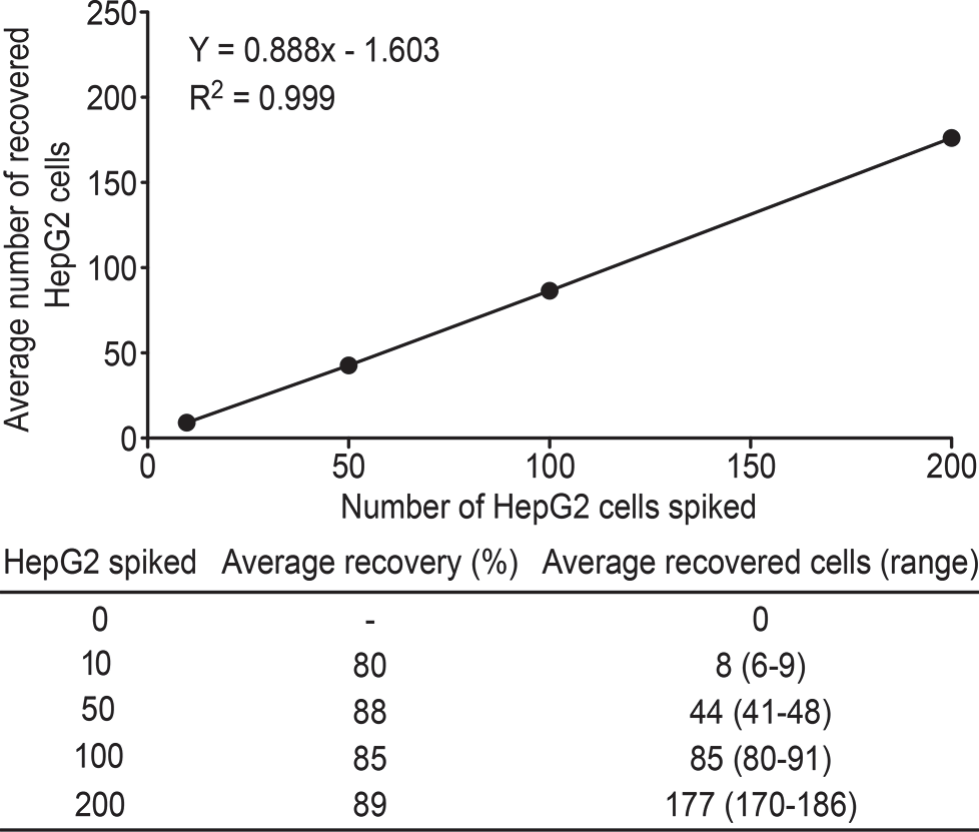
Calibration curve obtained using the optimized CanPatrol CTC enrichment technique in the spiking experiment (n = 8) using HepG2 cells at different dilutions.

The average recovery at each dilution of cells was at least 80% and ranged from 80% to 89% (Fig 2).

### Efficacy of the CanPatrol CTC enrichment technique: before vs after optimization

To compare the efficacy of the two methods for CTC isolation and characterization, 18 samples were tested. For each sample, 5 ml of blood was applied for CTC isolation and characterization of each method. The results are shown in Table 4. It has been shown that a greater number of CTCs was detected in 5 ml of blood after optimization. For the “before optimization” group, some atypical cells were found in samples #2, #5, #6, #12, #13, #14, #16 and #17 that were probably unlabeled CTCs. Blood samples #7, #10 and #18 were viscous, and the loss of CTCs from these samples when using the method without optimization was probably due to the multiple centrifugation and washing steps.

**Table 4:** Comparison of the efficacy of the CanPatrol CTC enrichment technique before and after optimization.

#	Cancer type	Clinical stage	CanPatrol^TM^ CTC Enrichment Technique							
			Before optimization				After optimization			
			Epithelial CTCs	Biophenotypic epithelial/mesenchymal CTCs	Mesenchymal CTCs	Total number of CTCs	Epithelial CTCs	Biophenotypic epithelial/mesenchymal CTCs	Mesenchymal CTCs	Total number of CTCs
1	NSCLC	T3N0M0	0	0	0	0	0	1	0	1
2		T3N2M0	1	1	0	2	2	2	0	4
3			0	0	0	0	0	0	0	0
4			0	0	1	0	0	0	3	3
5			1	0	0	1	1	4	0	5
6		T2N2M1	2	2	0	4	5	10	0	15
7			0	2	1	3	0	7	3	10
8			0	0	0	0	0	0	0	0
9			0	1	0	1	0	1	0	1
10			2	0	0	2	4	5	0	9
11	Breast cancer	T2N1M0	0	0	0	0	0	0	0	0
12			0	0	2	2	0	0	7	7
13			0	1	0	1	0	1	4	5
14			0	5	0	5	0	8	0	8
15			0	0	0	0	0	0	0	0
16		T3N2M1	0	3	3	6	1	5	7	13
17			1	1	0	2	1	7	0	8
18			0	0	1	1	0	1	10	11

### Further validation of the optimized CanPatrol CTC enrichment technique using clinical samples

**Fig 3:**
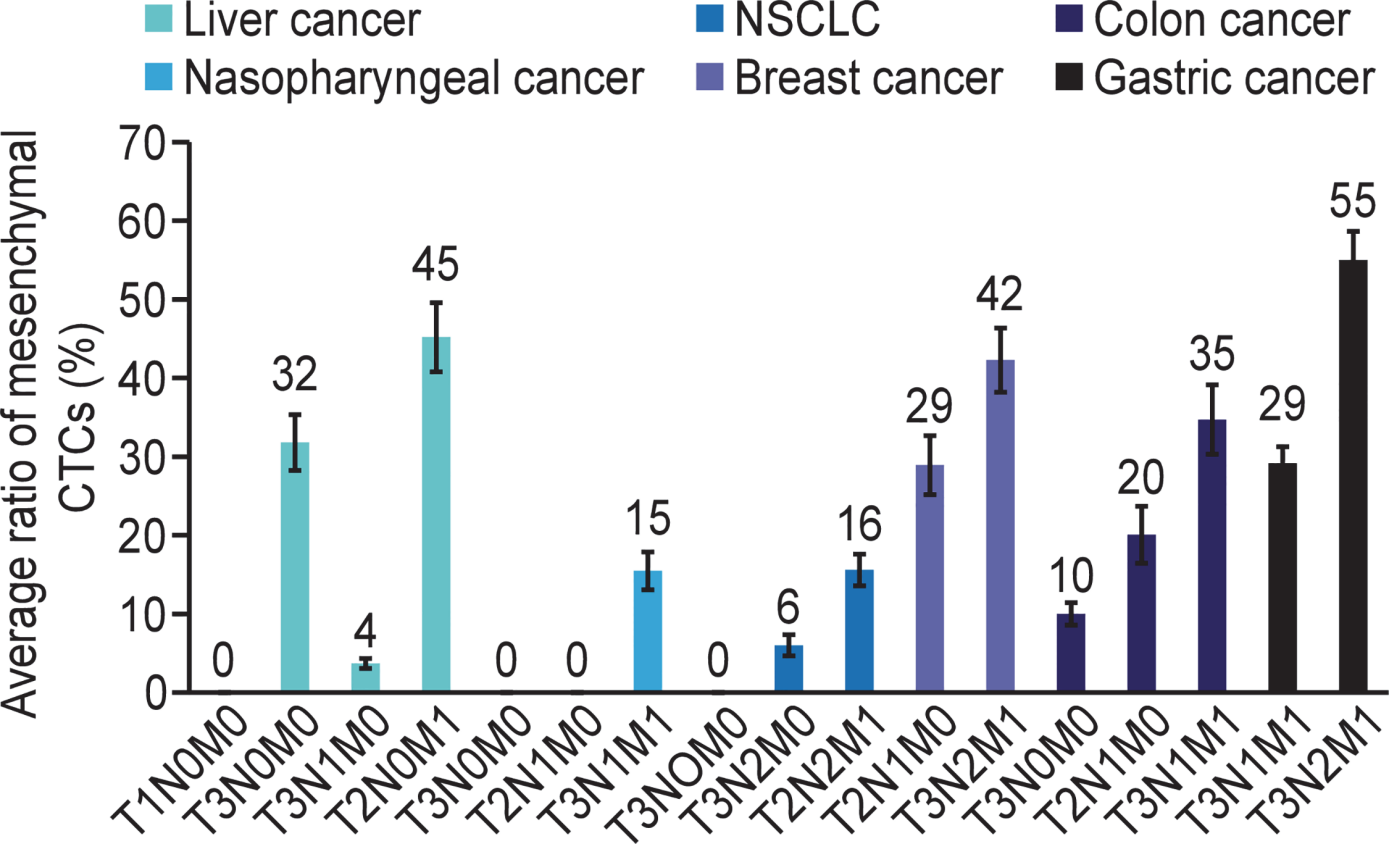
The average ratio of mesenchymal CTCs in each positive sample in cancers at different stages. Compared with the earlier stages of cancer, the average ratio of mesenchymal CTCs in each positive sample increased in the metastatic stages of cancer. The error bars indicate standard deviations.

**Fig 4:**
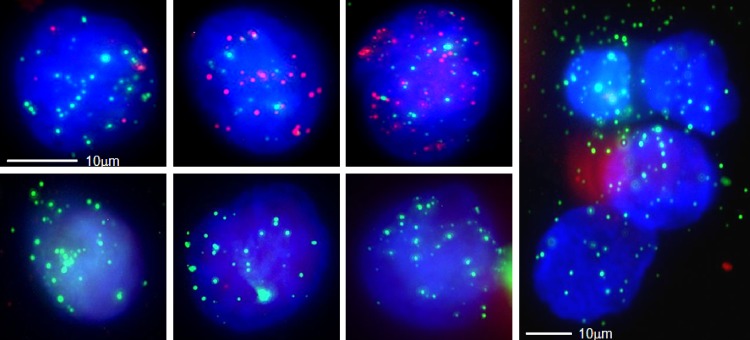
CTCs detected in a blood sample from a liver cancer patient. A total of 10 CTCs were detected in this sample; 3 single migratory biophenotypic epithelial/mesenchymal CTCs, 3 single migratory mesenchymal CTCs and a tumor microembolus containing 4 mesenchymal CTCs were observed (epithelial biomarkers are indicated by red fluorescence; mesenchymal biomarkers are indicated by green fluorescence).

A total of 164 blood samples from patients with NSCLC or liver, nasopharyngeal, breast, colon or gastric carcinoma (29 with NSCLC, 40 with liver cancer, 24 with nasopharyngeal cancer, 18 with breast cancer, 38 with colon cancer, and 15 with gastric cancer) were collected for CTC isolation and characterization. The results demonstrated that CTCs were detected in 107(65%) of 164 blood samples; of the CTC-positive samples, 24(60%), 14(58%), 12(67%), 24(63%), 10(67%), and 23(79%) were from liver cancer, nasopharyngeal cancer, breast cancer, colon cancer, gastric cancer, and NSCLC patients, respectively (Table 5). The median number of CTCs increased in the metastatic stages of the different types of cancer. The CTCs were classified into three subpopulations according to the EMT markers applied in this study, including epithelial CTCs, biophenotypic epithelial/mesenchymal CTCs, and mesenchymal CTCs. In the metastatic stages of the different types of cancer, such as T3N1M1 and T3N2M1, a greater proportion of samples contained mesenchymal CTCs (Table 5). The results also indicated that the average ratio of mesenchymal CTCs in each positive sample increased in the later stages of cancer compared with the earlier stages of cancer (Fig 3). Circulating tumor microemboli (CTM) with a mesenchymal phenotype were detected in three blood samples from patients in the metastatic stages of cancer (Table 6), including one liver cancer patient at T3N1M1 (Fig 4), one nasopharyngeal cancer patient at T3N1M1, and one breast cancer patient at T3N2M1. CTM were defined as multicellular CTC clusters containing greater than or equal to 4 cells [7].

**Table 5:** CTCs detected in patients with NSCLC or liver, nasopharyngeal, breast, colon or gastric cancers.

Cancer type	Clinical stage	Number of blood samples	Number of positive samples (%)	Number of positive samples containing CTM (%)	Median number of CTCs	Range of CTC count	Classification of CTCs		
							Number of samples containing epithelial CTCs (%)	Number of samples containing biophenotypic epithelial/mesenchymal CTCs (%)	Number of samples containing mesenchymal CTCs (%)
Liver cancer	T1N0M0	11	4(36%)	0(0%)	0	0–4	3(27%)	2(18%)	0(0%)
	T3N0M0	14	9(64%)	0(0%)	3	0–12	4(29%)	8(57%)	6(43%)
	T3N1M0	5	3(60%)	0(0%)	1	0–9	2(40%)	2(40%)	1(20%)
	T3N1M1	10	8(80%)	1(10%)	8	0–21	3(30%)	6(60%)	5(50%)
Nasopharyngeal cancer	T3N0M0	5	2(40%)	0(0%)	0	0–2	1(20%)	1(20%)	0(0%)
	T2N1M0	10	5(50%)	0(0%)	1	0–5	3(30%)	4(40%)	0(0%)
	T3N1M1	9	7(78%)	1(11%)	2	0–9	6(67%)	5(56%)	2(22%)
NSCLC	T3N0M0	3	2(67%)	0(0%)	1	0–3	1(33%)	1(33%)	0(0%)
	T3N2M0	6	5(83%)	0(0%)	3	0–5	2(33%)	4(67%)	1(17%)
	T2N2M1	20	16(80%)	0(0%)	4	0–15	8(40%)	15(75%)	5(25%)
Breast cancer	T2N1M0	12	7(58%)	0(0%)	4	0–8	0(0%)	6(50%)	4(33%)
	T3N2M1	6	5(83%)	1(17%)	11	0–30	4(67%)	4(67%)	3(50%)
Colon cancer	T3N0M0	8	2(25%)	0(0%)	0	0–5	0(0%)	2(25%)	1(13%)
	T2N1M0	20	13(65%)	0(0%)	1	0–7	5(25%)	10(50%)	4(20%)
	T3N1M1	10	9(90%)	0(0%)	4	0–12	1(10%)	7(70%)	5(50%)
Gastric cancer	T3N1M1	7	4(57%)	0(0%)	1	0–15	0(0%)	4(57%)	3(43%)
	T3N2M1	8	6(75%)	0(0%)	4	0–45	2(25%)	5(63%)	5(63%)
Total		164	107(65%)	3(2%)	2	0–45	45(27%)	86(52%)	45(27%)

**Table 6:** The three blood samples containing CTM from liver, nasopharyngeal and breast cancers.

Cancer types	Clinical stage	The number of CTM in each sample	The number of CTCs in each CTM	CTM phenotype
Liver cancer	T3N1M1	1	4	mesenchymal phenotype
Nasopharyngeal cancer	T3N1M1	1	5	mesenchymal phenotype
Breast cancer	T3N2M1	1	7	mesenchymal phenotype

## Discussion

Accumulating evidence has indicated that CTCs can be used as a biomarker to non-invasively monitor cancer progression and provide information to guide the choice of therapy [24]. Different techniques have been reported for CTC isolation and characterization, which are based on the physical properties of CTCs or cell surface antigens. However, the isolation and detection of CTCs are significantly hampered by the phenotypic alterations that are common to CTCs. Previous studies have shown that epithelial antigen-based approaches may fail to detect the most aggressive CTC subpopulation, which may have undergone EMT [25]. EMT is a multistep process that plays a key role in metastasis and cancer progression, and CTCs bearing characteristics of an EMT phenotype are presumed to be involved in tumor dissemination and metastasis. Therefore, CTC detection methods require optimization by including biomarkers that are not repressed during the EMT process.

In this study, we applied the optimized CanPatrol CTC enrichment technique for CTC isolation and characterization. This technique includes two major steps: a filter-based method to isolate CTCs and subsequent characterization of the CTCs using EMT markers, including the epithelial markers EpCAM and CK and the mesenchymal markers vimentin and twist. We chose these biomarkers for CTC characterization, because EpCAM and CK are commonly used for epithelial CTC detection, and previous studies have demonstrated that the expression of the mesenchymal markers twist or vimentin in CTCs is associated with cancer metastasis [22]. Compared with the CellSearch platform, which uses anti-EpCAM-coated magnetic beads to capture CTCs, the optimized CanPatrol CTC enrichment technique is an unbiased CTC isolation method that allows for the isolation of CTCs not expressing epithelial antigens, such as EpCAM. Our results showed that brain glioma cells, such as the cell line U118MG lack EpCAM expression, and cannot be isolated using the CellSearch platform. However, these tumor cells can be easily isolated and characterized using the optimized CanPatrol CTC enrichment technique, as it is a filter-based method that uses a cocktail of epithelial and mesenchymal markers to characterize the tumor cells. In the pre-optimization method, CTC isolation was based on red blood cell lysis to remove erythrocytes. Erythrocyte removal was followed by depletion of CD45+ leukocytes using a magnetic bead separation method, and CTCs were subsequently isolated by virtue of their larger size (filter-based) compared with leukocytes. The advantage of this technique was that 99.98% of leukocytes were depleted and a lower number of leukocytes remained on membrane, making it easier to observe the CTCs under a microscope. However, when the sample size was expanded to validate this method, two issues arose. First, the blood of some cancer patients was viscous, and multiple centrifugation and washing steps led to the loss of CTCs. Second, the low sensitivity of traditional immunostaining method might fail to detect some CTCs that express low levels of the target proteins. Compared to the previous method, the optimized method is more suitable for CTC enumeration and characterization. First, the CTC isolation steps are simpler, and the fewer centrifugation and washing steps help to enhance CTC enrichment. Second, an RNA-ISH method combined with a branched DNA signal amplification technology was used to label the isolated CTCs. Compared with the immunostaining method, this method has the advantages of high sensitivity and background suppression. When we compared the efficacy of the CanPatrol CTC enrichment technique before and after optimization, the results indicated that a greater number of CTCs was detected in 5 ml of blood after optimization. To further validate the optimized CanPatrol CTC enrichment technique, 164 blood samples from six different types of cancer patients were tested. CTCs were detected in 107(65%) blood samples, and 0–45 CTCs were found in each sample. The CTCs could be classified into three subpopulations according to the EMT markers that they expressed, including epithelial CTCs, biophenotypic epithelial/mesenchymal CTCs, and mesenchymal CTCs. Our study showed that mesenchymal CTCs were more common to be found in metastatic stages of cancer. The average ratio of mesenchymal CTCs in each positive sample increased in the metastatic stages of cancer compared with the earlier stages of cancer. CTM with a mesenchymal phenotype were also detected in the metastatic stages of cancer. CTM are tumor cell clusters and are associated with high metastatic potential [7]. Our findings are consistent with previous reports indicating that mesenchymal CTCs are associated with metastasis and disease progression [7].

In summary, compared with before optimization, the optimized CanPatrol CTC enrichment technique is more effective for CTC isolation and characterization. The presence of the EMT phenotype was demonstrated in the CTCs of a variety of cancers, including NSCLC and liver, nasopharyngeal, breast, colon and gastric cancers. Because EMT can be used as a potential biomarker of cancer metastasis and therapeutic resistance, the classification of CTCs according to their EMT phenotype helps identify the most aggressive CTC subpopulation and provides data for clinical applications.

## Conclusion

In conclusion, by using EMT markers, the optimized CanPatrol CTC enrichment technique is able to classify CTCs into three subpopulations: epithelial CTCs, biophenotypic epithelial/mesenchymal CTCs, and mesenchymal CTCs. This technique is suitable for a broad range of carcinomas.
